# A review on slip boundary conditions at the nanoscale: recent development and applications

**DOI:** 10.3762/bjnano.12.91

**Published:** 2021-11-17

**Authors:** Ruifei Wang, Jin Chai, Bobo Luo, Xiong Liu, Jianting Zhang, Min Wu, Mingdan Wei, Zhuanyue Ma

**Affiliations:** 1 Shaanxi Key Laboratory of Well Stability and Fluid & Rock Mechanics in Oil and Gas Reservoirs, College of Petroleum Engineering, Xi’an Shiyou University, 710065, China; 2 Research Institute of Exploration and Development, Zhongyuan Oilfield Company, SINOPEC, Puyang 457001, China

**Keywords:** boundary condition, interfacial properties, nanofluidics, slip length, unconventional reservoirs

## Abstract

The slip boundary condition for nanoflows is a key component of nanohydrodynamics theory, and can play a significant role in the design and fabrication of nanofluidic devices. In this review, focused on the slip boundary conditions for nanoconfined liquid flows, we firstly summarize some basic concepts about slip length including its definition and categories. Then, the effects of different interfacial properties on slip length are analyzed. On strong hydrophilic surfaces, a negative slip length exists and varies with the external driving force. In addition, depending on whether there is a true slip length, the amplitude of surface roughness has different influences on the effective slip length. The composition of surface textures, including isotropic and anisotropic textures, can also affect the effective slip length. Finally, potential applications of nanofluidics with a tunable slip length are discussed and future directions related to slip boundary conditions for nanoscale flow systems are addressed.

## Introduction

A basic postulate in the study and design of macroscopic fluidic systems based on the knowledge of fluid mechanics is that the no-slip boundary condition is valid at the solid–liquid interface [1]. In the last two centuries, this no-slip boundary condition has been successfully applied to solve problems associated with macroscopic flows [2–5]. However, in the field of fluid transport at the micro-/nanoscale, the problem is not that simple and a possible deviation from the classical hypothesis may take place, resulting in liquid slippage at solid surfaces [6–9]. In addition, as downsizing can result in an increased surface-to-volume ratio, the solid–liquid interfacial properties, such as wettability and surface roughness, become key factors in the determination of liquid properties near the interface of nanosized systems, and may dramatically affect the slip flow boundary conditions [10–15]. For example, many studies have shown that on hydrophobic surfaces, roughness may lead to a transition to a superhydrophobic state, significantly lowering the ability of liquid drops to stick. In other words, liquids can easily slip along such solid surfaces and the no-slip boundary condition is no longer valid. The slip length, a quantity that reflects the amount of slip at a given surface, can reach orders of magnitude of many microns [13].

The impetus to investigate flow boundary conditions, which are valid in nanoscale fluidic systems, lies in the potential applications in many areas of applied science and engineering [16–21]. In the field of nanofluidics, probing flow boundary conditions for nanoconfined liquids contributes to the deep understanding of the nature of nanohydrodynamics, which is the theoretical basis for the design and fabrication of nanofluidic devices. In addition, understanding the slip flow behavior in nanoporous media is also of great significance in the field of development of shale reservoirs [22]. The shale oil transport could be enhanced due to the positive slip length compared with that of the no-slip transport model [16]. Moreover, increasing the slip length can also raise the energy conversion efficiency from mechanical to electrical energy of the nanofluidic devices due to the reduction of flow resistance. For instance, it was shown that a slip length of 50 nm could increase the efficiency of energy conversion from 3 to 70% [23].

Generally, the methods to investigate slip boundary conditions for nanoconfined liquids include theoretical analysis, physical experiments, and numerical simulations [8,24–34]. In recent years, machine learning methods have also been applied in the study of dynamic properties of liquids including diffusion and slip flow behavior, and in the prediction of the slip length at the nanoscale [35–37]. Owing to the rapid development of technology, experimental studies of flow boundary conditions have been successfully extended to nanoscale systems [8,34,38–39]. For example, based on surface force apparatus (SFA) and atomic force microscopy (AFM) measurements, many researchers have investigated the slippage characteristics of nanoconfined liquid flows and derived the slip length according to its correlation with the hydrodynamic force [39–42]. However, compared with experimental methods, numerical simulations, such as the lattice Boltzmann method and molecular dynamics (MD) simulation, are more attractive in many aspects. First, numerical simulations can readily reach the system sizes and timescales of practical nanoflows [43]. Additionally, numerical methods can provide a controllable way to change a certain property of liquid or solid walls while other properties remain unchanged [44]. In comparison with physical experiments, numerical simulations allow researchers to study the density, velocity profiles, and other properties with a high resolution [43]. Finally, when the flow systems are under extreme conditions, such as at high shear rates, it has also been proven that numerical simulations are more efficient than experimental methods [45]. Therefore, in this review we mainly focus on the numerical investigations and theoretical analysis on slip boundary conditions for nanoscale liquid transport.

This review is organized as follows. First, the concepts and categories of slip length are presented. Then, the effects of different interfacial properties on slip length are analyzed. Next, the potential applications of nanofluidics with tunable slip length are discussed. Finally, the conclusions are drawn and possible future directions about slip boundary conditions for nanoscale fluid flow are prospected.

## Review

### 1 Background

#### 1.1 Definition of slip length and its microscopic expression

**Figure 1:**
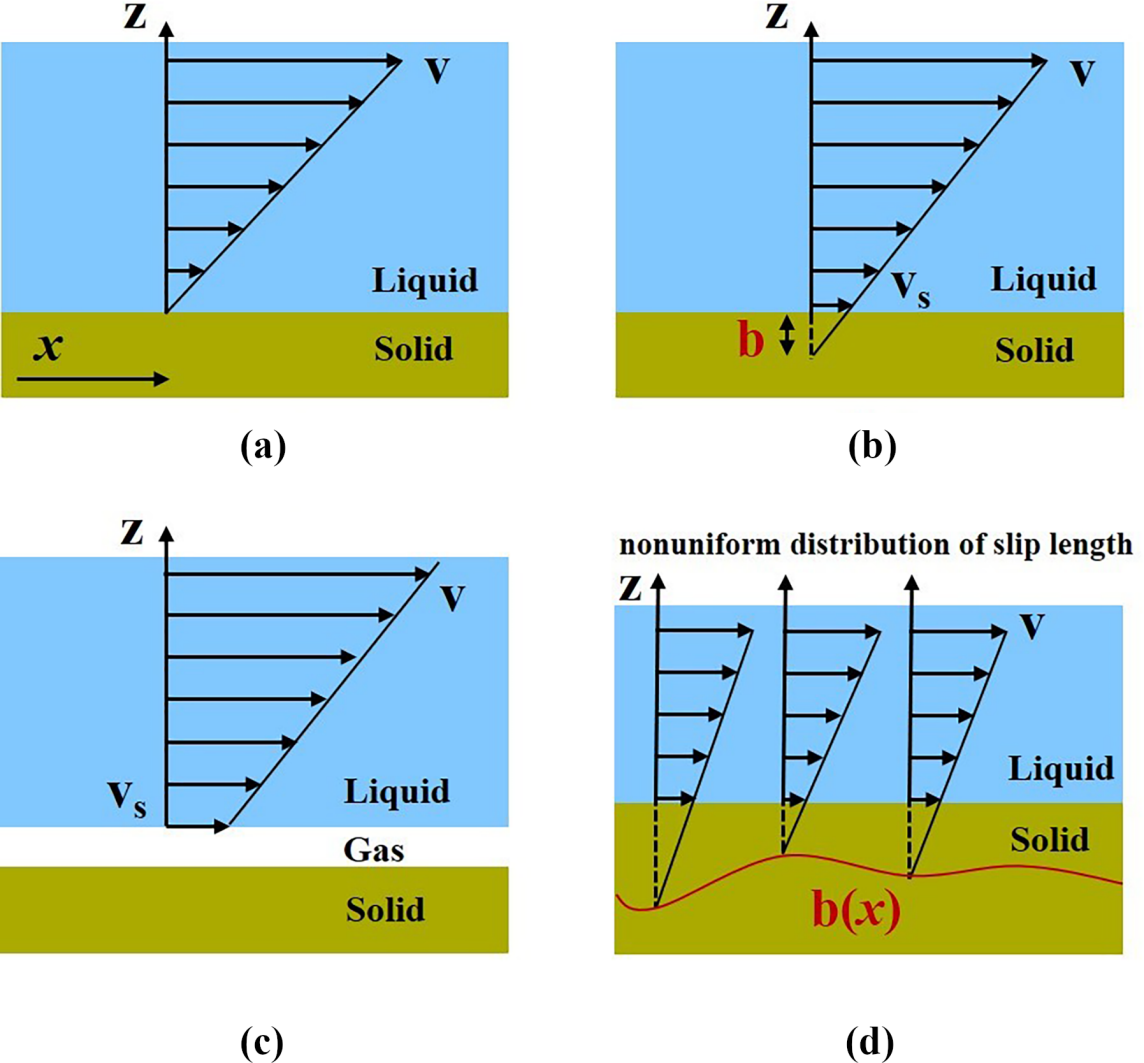
Schematic representation of the definition of slip lengths: (a) no-slip, (b) true slip length, (c) apparent slip length, and (d) effective slip length. Figures 1a–c were redrawn from [49] and Figure 1d was redrawn from [50].

As illustrated in Figure 1a, it is assumed in the the no-slip boundary condition that the layer of liquid adjacent to the solid surface moves with the same velocity as the solid surface. It has been demonstrated that the classical no-slip boundary condition holds true for numerous cases of macroscopic experiments with no microscopic description. In 1823, Navier proposed the partial slip boundary condition, and the concept of slip length *b* was introduced to reflect the amount of liquid slip at a given surface. The slip length is the distance beyond the solid–liquid interface where the liquid velocity linearly extrapolates to zero (see Figure 1a). The relationship between slip length *b* and liquid slip velocity ν_s_ at the surface can be expressed as follows:


[1] $$\nu_{\text{s}}=b\cdot \frac{\partial \nu }{\partial h},$$

where ν is liquid velocity and *h* is the channel height.

When the liquid is at equilibrium, the viscous shear stress 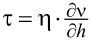 is exerted by the liquid on the wall, where η is the shear viscosity of the liquid, equal to the friction stress suffered by the liquid from the wall, which is expressed as σ = λν_s_, where λ represents the interfacial friction coefficient [46]. Therefore, the slip length can be expressed as 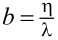, which indicates that the slip length is reduced with the increase in the friction between liquid and solid surfaces. Since the interfacial friction coefficient can be expressed in terms of the Green–Kubo expression, the microscopic expression of slip length can be written as follows [1,46]:


[2] $$b=\frac{\eta }{\lambda }=\frac{\eta Sk_{\text{B}}T}{\int_{0}^{\infty }\langle F_{l}\left(t\right)F_{l}\left(0\right)\rangle \text{d}t}$$

where *S* is the area of the solid wall and *F*_l_ is the component of the instantaneous force that the solid wall exerts on the liquid in the tangent direction.

#### 1.2 True slip, apparent slip, and effective slip

Typically, the local slip length can be divided into two categories including true slip length and apparent slip length [47–48]. True slip means that the liquid molecules are essentially sliding along the solid surfaces at a molecular level, as illustrated in Figure 1b. Its value cannot be negative. On the other hand, apparent slip means that the liquid slips at the surface of a thin fluid film of a different phase near the solid wall. That is, the liquid slippage occurs at the liquid–liquid or liquid–gas interface rather than at the liquid–solid interface, as shown in Figure 1c. It should be noted that the value of the apparent slip length can be positive or negative. For a negative apparent slip length, or negative slip length for short, the no-slip boundary condition holds at the liquid–liquid interface with a thin immobile liquid layer in the vicinity of the solid surface.

In practice, however, the actual flow system is complex owing to the heterogeneous and rough surfaces, or to the existence of gas bubbles. Therefore, the slip length may be nonuniformly distributed. In order to determine the flow boundary condition in such complex systems, the concept of effective slip length has been introduced [51–55]. The effective slip length is actually the equivalent property, based on a hypothetical and homogeneous flow channel, which yields the same flow rate as that of the complex system under the same ambient conditions and external perturbations [56].

### 2 Effects of interfacial properties on slip length

#### 2.1 Surface wettability effects

**Figure 2:**
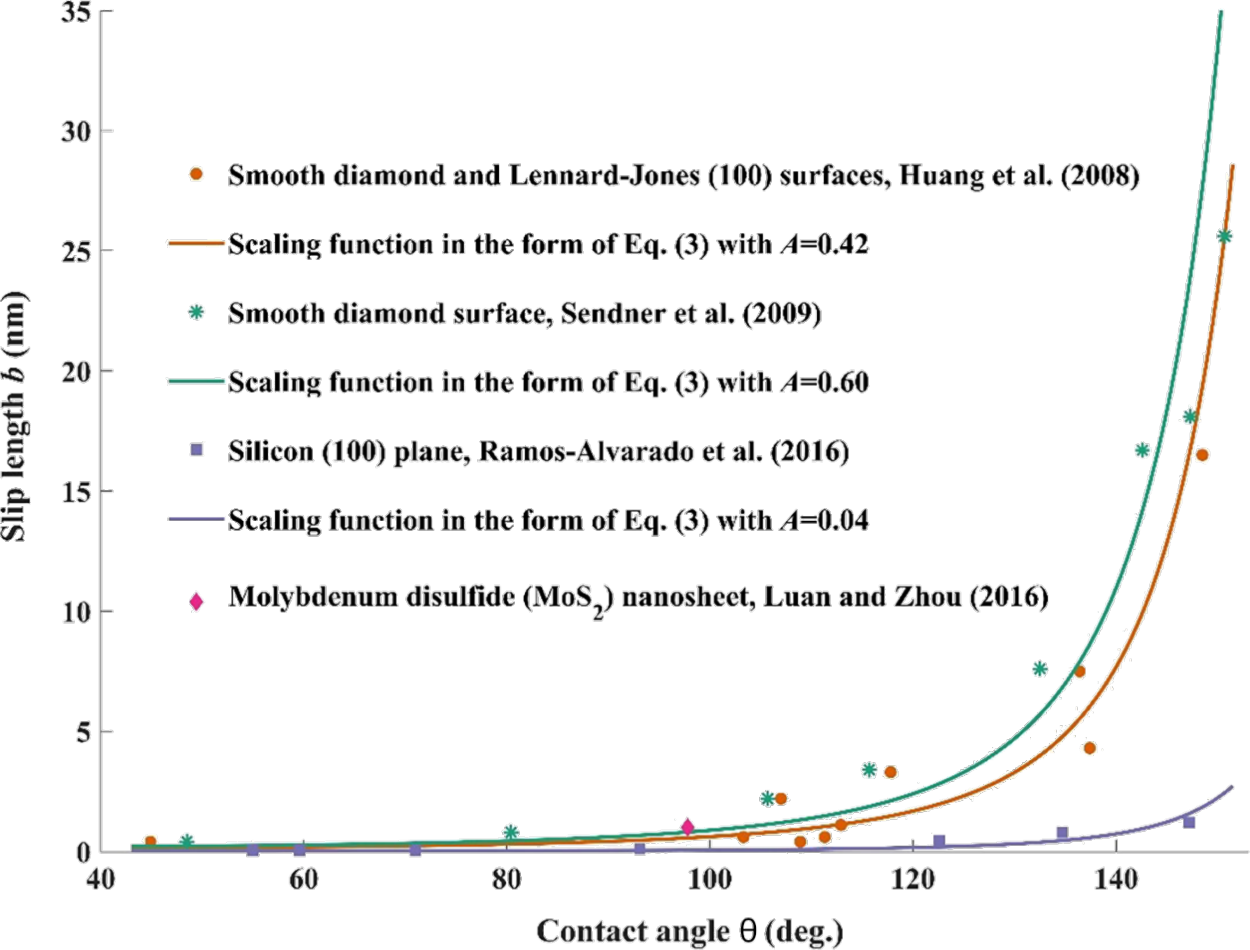
Variation of slip length with static contact angle for water flow on different types of smooth surfaces. The graph depicted in Figure 2 was redrawn from data in [62–65].

**2.1.1 Variation of positive slip length.** It is intuitive that the wettability of a liquid on solid surfaces could affect the boundary slip, and many previous investigations have shown that, from qualitative points of view, the positive slip length monotonically increases with the increase in the contact angle [57–61]. Furthermore, when studying the water flow on smooth surfaces, there is a quasiuniversal relationship between the slip length and the static contact angle as follows (see Equation 3 and Figure 2).


[3] $$b=A\cdot {\left(1+\cos\theta \right)}^{-2}$$

It has been shown that Equation 3 can be interpreted on the grounds of definite physical principles, according to the microscopic connection between slip length, contact angle, and the liquid–solid interaction parameter [62].

**Figure 3:**
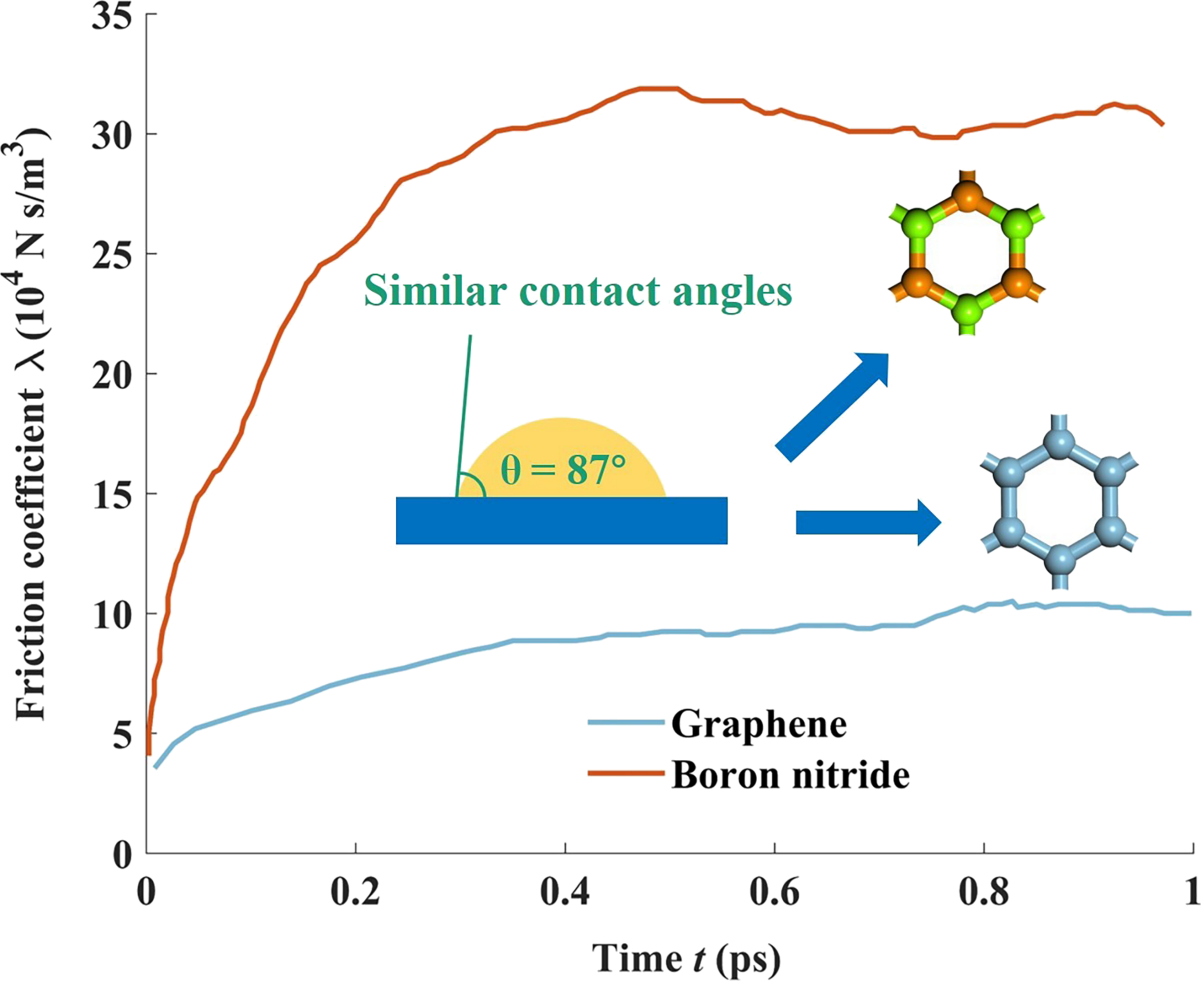
Comparison between the friction coefficients of water on graphene and boron nitride surfaces. The inset illustrates similar contact angles (around 87°) of water on both surfaces. Figure 3 was redrawn from [69].

However, it should be noted that this model is only applicable to cases of water slippage on smooth surfaces, and there are some deviations for water slippage on rough surfaces [66]. On the other hand, even on very smooth surfaces, the contact angle, surface–water interaction energy, and water slippage (or friction coefficient) may also not have a one-to-one correspondence between each other [67–69]. Contrary to the conventional wisdom, where slip boundary conditions are not valied for water slippage on hydrophilic surfaces, some simulation observations show that liquid water can still slip even when the attraction between water and the solid wall is strong [67–68]. Besides the solid–water interaction energy, water slippage is also determined by the spatial distribution of water molecules within the contact layer on solid surfaces [68–69]. Under the condition of same water–solid interaction energy, for the case with more uniform and more compact distribution of water molecules near solid surfaces, liquid water can slip more easily. Otherwise, for the case with more isolated and corrugated distribution, the migration of liquid water molecules is more limited, thus leading to the reduction of water slippage. Figure 3 shows that although water shows similar wetting properties on surfaces of boron nitride and graphene, the friction coefficient (or slip length) of water on boron nitride is much larger (or lower) than that on graphene due to a more corrugated distribution of water molecules on the surface of boron nitride [69].

**Figure 4:**
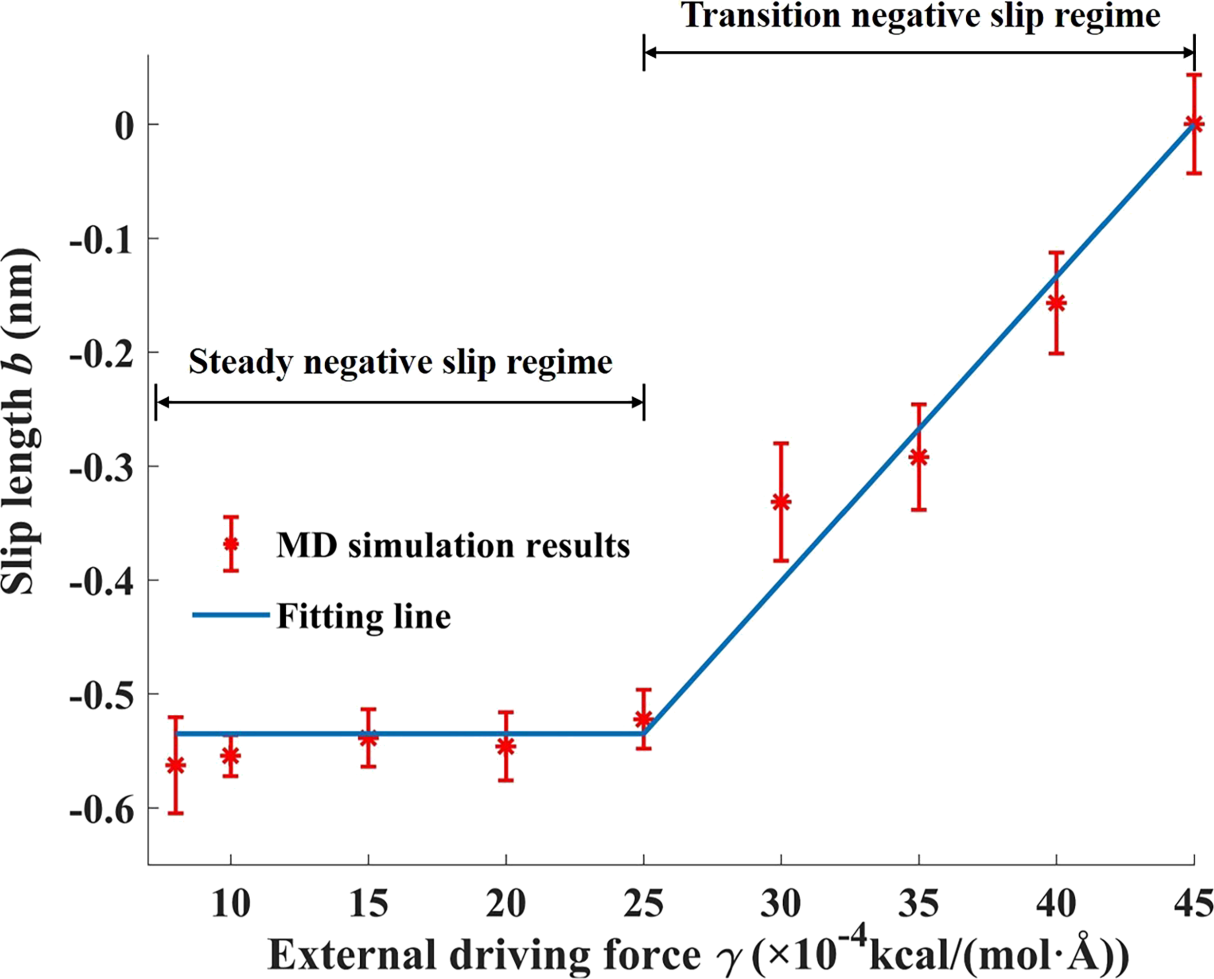
Variation of negative slip length values with an external driving force. The uncertainty of the MD simulation results come from the noise of the data points. Figure 4 was redrawn from [72].

**2.1.2 Variation of negative slip length.** As stated in Section 1.2, the negative slip length means that there is a static liquid film near solid surfaces. It was shown by many previous studies that a negative slip length may exist when liquid flows on a strong hydrophilic surface [34,57,70]. Although the value of negative slip length is typically of the order of one or several molecular diameter sizes, it can play a crucial role especially in nanometric flow systems [34,71]. Most of the previous studies dealt with the negative slip phenomena by treating the immobile fluid layer as a solid wall and applying no-slip boundary conditions at the shifted boundary [34,70]. However, they just regarded the thickness of the immobile fluid layer as constant and barely focused on the variation of the negative slip length (for instance, with external perturbations) in spite of the critical importance they may have on nanoscale fluid transport behavior. Zhang et al. found that the negative slip length exists due to the superhydrophilic nature of the solid wall and also investigated the variation of the negative slip length with an external driving force [72], which represents the pressure difference for the Poiseuille flow (the results are shown in Figure 4). Figure 4 shows that two regimes are identified to characterize the variation of negative slip length values with an external driving force. When the external driving force is relatively small, the negative slip length is nearly constant, and this regime is called steady negative slip regime. The second regime is called transition negative slip regime, where the negative slip length linearly decreases to zero with an external driving force. It should be noted that this conclusion was drawn based on an ideal Lennard–Jones fluid. Its applicability on other Newtonian fluids with properties such as polarity and even on non-Newtonian fluids should be further validated.

#### 2.2 Surface charge effects

**Figure 5:**
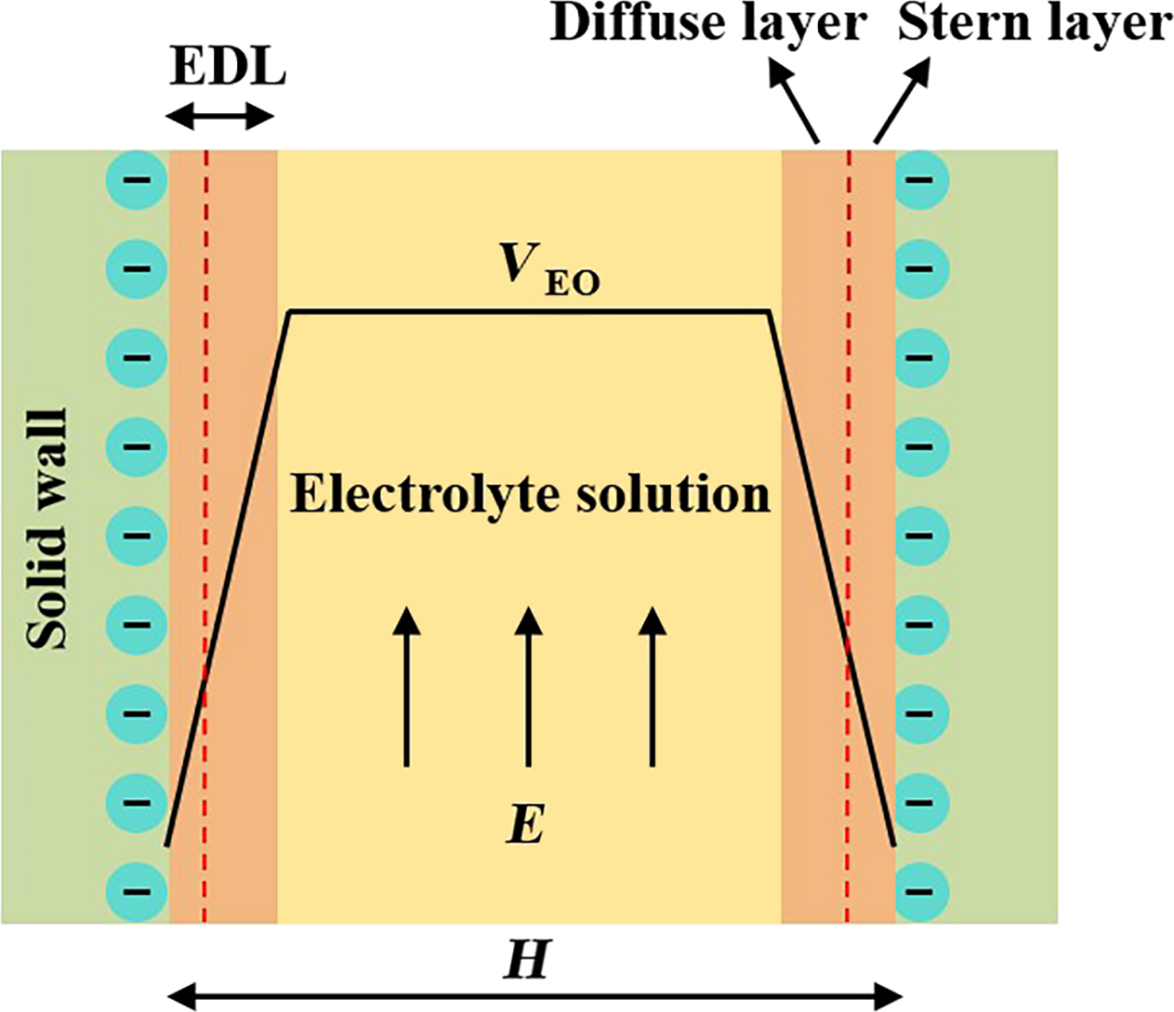
Electrical double layer (EDL) model at the solid–liquid interface and the plug-like shape of the velocity profile for the electro-osmotic flow when the thickness of the EDL is much smaller than the channel width *H*.

Investigating electrokinetics phenomena, including electro-osmosis, electrophoresis, streaming current or potential, and sedimentation potential have played crucial roles in the development of microfluidics and nanofluidics [73–75]. Among them, the electro-osmotic flow refers to the motion of an electrolyte solution relative to the stationary charged surface due to an applied electric field. Due to the surface charge on the solid surface, an electrical double layer (EDL), which is comprised of oppositely charged ions, naturally forms within the liquid in the vicinity of the solid surface. As shown in Figure 5, the EDL consists of an immobile Stern layer and a mobile diffuse layer. Although the extension of the EDL is typically on the order of a few nanometers, it has a significant impact on the electro-osmotic flow behavior [46,76–79]. In the classical theories, the electro-osmotic flow is described based on the no-slip boundary condition at the solid–liquid interface, and when the thickness of the EDL is much smaller than the characteristic geometrical length, the shape of the velocity profile appears to be plug-like. Nonetheless, many studies have shown that on hydrophobic solid surfaces, the ionized solution driven by an external electric field may experience a slippage [80]. In addition, the hydrodynamic slippage at the solid–liquid interface can in turn largely enhance the electro-osmotic velocity, which increases the urgency for a deep research on the liquid slippage phenomena in electrokinetics.

Owing to the direct influence of surface charge density on the distribution of the electrostatic potential within the EDL, many previous studies have investigated how the surface charge density can affect the solvent hydrodynamic slippage on solid surfaces. It was demonstrated by many researches that when the surface charge density increases, the slip length correspondingly decreases [23,46]. Joly et al. proposed an approximate model to describe the relationship between the slip length and surface charge density. They validated the model by using a MD simulation with an ionized solution system represented by models of ideal solvents and ions [46]. Furthermore, Rezaei et al. performed MD simulations to study the electro-osmotic flow of an aqueous NaCl solution on a charged silicon surface [23], and similar conclusions as the ones proposed by Joly et al. were drawn in terms of the relationship between slip length and surface charge density. On the other hand, it should be noted that the increase in surface charge density can also directly increase the bare surface potential, thus increasing the zeta potential, denoted by ζ, which is a key parameter that reflects the amplitude of the electrokinetic effects. Therefore, the increase in surface charge density can also lead to the enhancement of the electro-osmotic flow velocity, which indicates that there should be an optimum surface charge density in order to induce a maximum electro-osmotic flow velocity.

#### 2.3 Surface roughness effects

**Figure 6:**
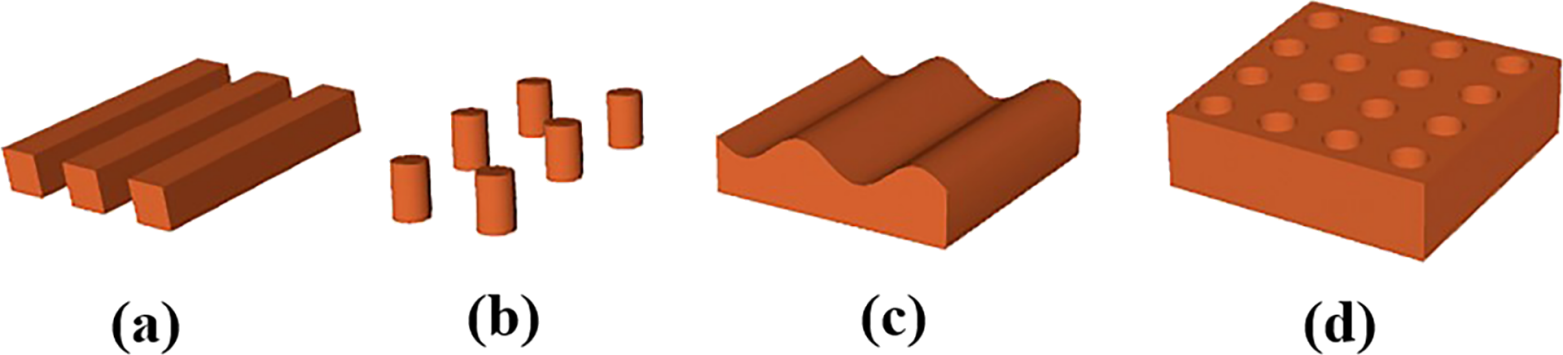
Representative roughness elements: (a) grooves, (b) pillars, (c) a rough surface with a sine/cosine relief, and (d) holes.

It is of great significance to investigate the effect of the surface roughness on the flow boundary conditions, since a perfectly smooth surface is an idealized model even at molecular scales. Numerous investigations have demonstrated that the variations of topography on the surface can drastically influence the effective slip length [81–82]. However, the challenges lie in the characterization of the rough surface owing to the random distribution of roughness elements on the actual surface [45]. Hence, most studies focus on the structured surfaces with well-defined roughness [42], and a series of roughness elements are periodically distributed on these surfaces as shown in Figure 6. Such structured surfaces, with definite dimensions of the structures, are very crucial for the design and fabrication of nanofluidic devices [83–85].

**Figure 7:**
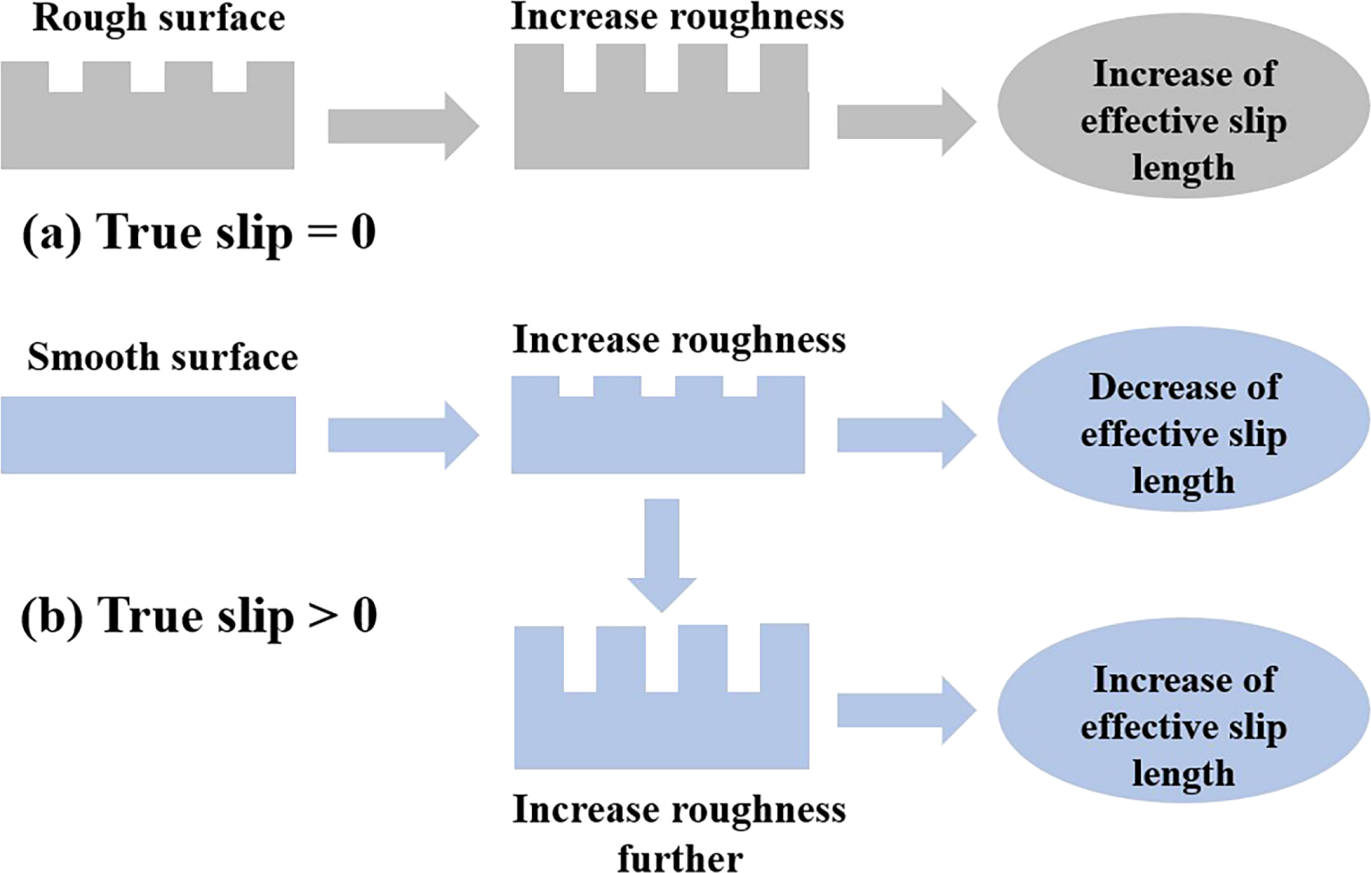
Variation of effective slip length with the amplitude of periodic structured surfaces when the (a) true slip length = 0 or (b) true slip length > 0. Figure 7 was redrawn from information given in [42,44,81,86–91].

Many previous works have shown that the topography variation of structured surfaces can induce the variation of the effective slip length [42,81,86–87]. For periodic structured surfaces, there is a general qualitative trend for the variation of the effective slip length with geometrical parameters of rough surfaces, such as amplitude. The influences of the amplitude on the effective slip length are illustrated in Figure 7. When the true slip length is zero, the increase of amplitude monotonically increases the effective slip length [81]. Nonetheless, when there is a true slip length, more complication is added to describe the relationship between the amplitude and effective slip length. It has been demonstrated by many studies that if the amplitude is comparable to or smaller than the local slip length, then an increase in amplitude leads to a reduction of the effective slip length [86–87]. Among those studies, MD simulations conducted by Yang show that compared with the fluid flow at smooth hydrophobic surfaces, where the true slip length is non-zero, the increase in amplitude leads to an increase in the drag resistance at the fluid–solid interface for rough surfaces of the same nature [86].

Interestingly, when the amplitude of the structured surface is large compared with the local slip length, the increase in amplitude enhances the liquid slippage on the surface, that is, the effective slip length increases. It was demonstrated by many researchers that if the surfaces are hydrophobic, the large increase in surface roughness may drastically increase the liquid slippage on the surface, even reaching the superhydrophobic state [44,88–91].

#### 2.4 Surface chemistry effects

A substantial number of studies have demonstrated that complex surfaces decorated with a sophisticated surface chemistry can dramatically affect the effective slip length for nanoscale flow systems. The surface textures we review here include isotropic and anisotropic textures. In addition, we also summarize the influence of gas bubbles on the liquid slippage, which is a common phenomenon in most experimental or numerical investigations.

**2.4.1 Isotropic textures.** Investigating how effective slip lengths emerge from isotropic textures with nonuniformly distributed slip lengths is of great significance due to the fact that many natural or synthetic materials are isotropic [49,92]. Such problems have been extensively studied by analytical or numerical methods [93–96]. Although the effective slip length for a general case cannot be deduced considering the underlying complex microstructures of isotropic surfaces, the approximate expressions for some limiting cases with simplified physics still gained much attention due to their acceptable accuracy and relatively low computational cost [93]. Of those, some investigations focused on the derivation of the prediction bounds for the effective slip length in some targeted cases [49,82]. For example, it has been demonstrated that the Hashin–Shtrikman upper and lower bounds can be applied to the effective slip length for a fluid flow on arbitrary two-component textures [49]. On the other hand, various approximate equations have been proposed to determine the effective slip lengths in terms of local slip lengths with given area fractions for common two-component textures. The expressions of effective slip lengths for some representative two-component textures are summarized in Table 1.

**Table 1:** Summary of approximate expressions of effective slip length for some representative two-component textures.

Shape	Structure schematic^a^	Expressions^b^

Stripes	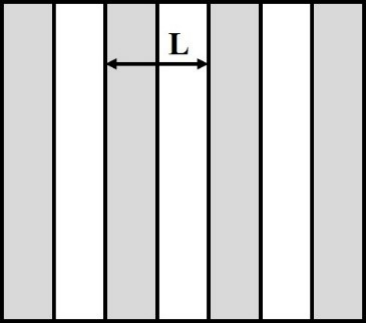	1. *b*_A_, *b*_B_ << *L*: *b*_e_ = ϕ_A_*b*_A_ + (1 − ϕ_A_)*b*_B_ [42,93] 2. *b*_B_ >> *b*_A_ >> *L*: 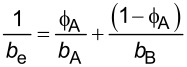 [93–95]
Chessboard	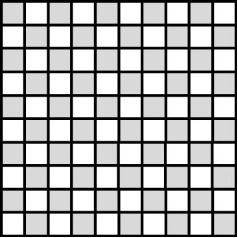	1. *b*_A_ = 0, *b*_B_ = *b*, and *b*/*H* << 1: *b*_e_ = *b*ϕ_B_ [49] 2. *b*_A_ = 0, *b*_B_ = *b*, and *b*/*H* >> 1: *b*_e_ = *H*/2 [49,96]
Posts	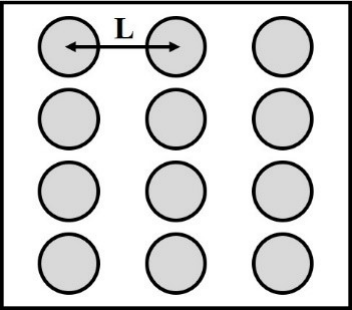	1. *b*_A_ = 0, *b*_B_ = ∞, and ϕ_B_ > 0.3: 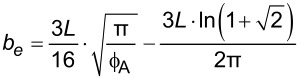 [97–98]
Holes	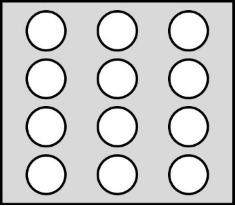	1. *b*_A_ = 0, *b*_B_ = ∞: 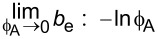 [98–99] For 0.22 < ϕ_A_ < 0.75: *b*_e_ = −0.134 ln(ϕ_A_) – 0.023 [100]
Holes (random distribution)	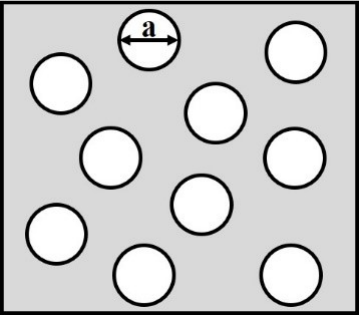	1. *b*_A_ = 0, *b*_B_ = ∞: 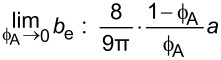 [101–102]

^a^Where the grey color represents component A and the white color represents component B. ^b^Where *b*_A_ and *b*_B_ are local slip lengths for a liquid flow on components A and B, respectively. ϕ_A_ and ϕ_B_ are area fractions of components A and B, respectively, and ϕ_A_ + ϕ_B_ = 1. *H* is the channel width.

**2.4.2 Anisotropic textures.** It is of great interest and importance to study and design directional structures owing to many potential applications, such as functional superwettable materials [103], water harvesting [104], and smog removal [105]. The directional structures induce a direction-dependent effective slip length, which can be quantified by the effective slip length tensor [13,47,56,106]. Although a large number of studies have investigated the effects of anisotropic textures on effective slip lengths, by means of numerical simulations and analytical solutions [13,107–110], a general expression, which can account for the variation of the effective slip length with the flow direction for arbitrary textures, is still too complicated to derive. Nonetheless, similar to the case of isotropic textures, as stated in Section 2.4.1, approximate equations for some limiting cases have been proposed.

**Figure 8:**
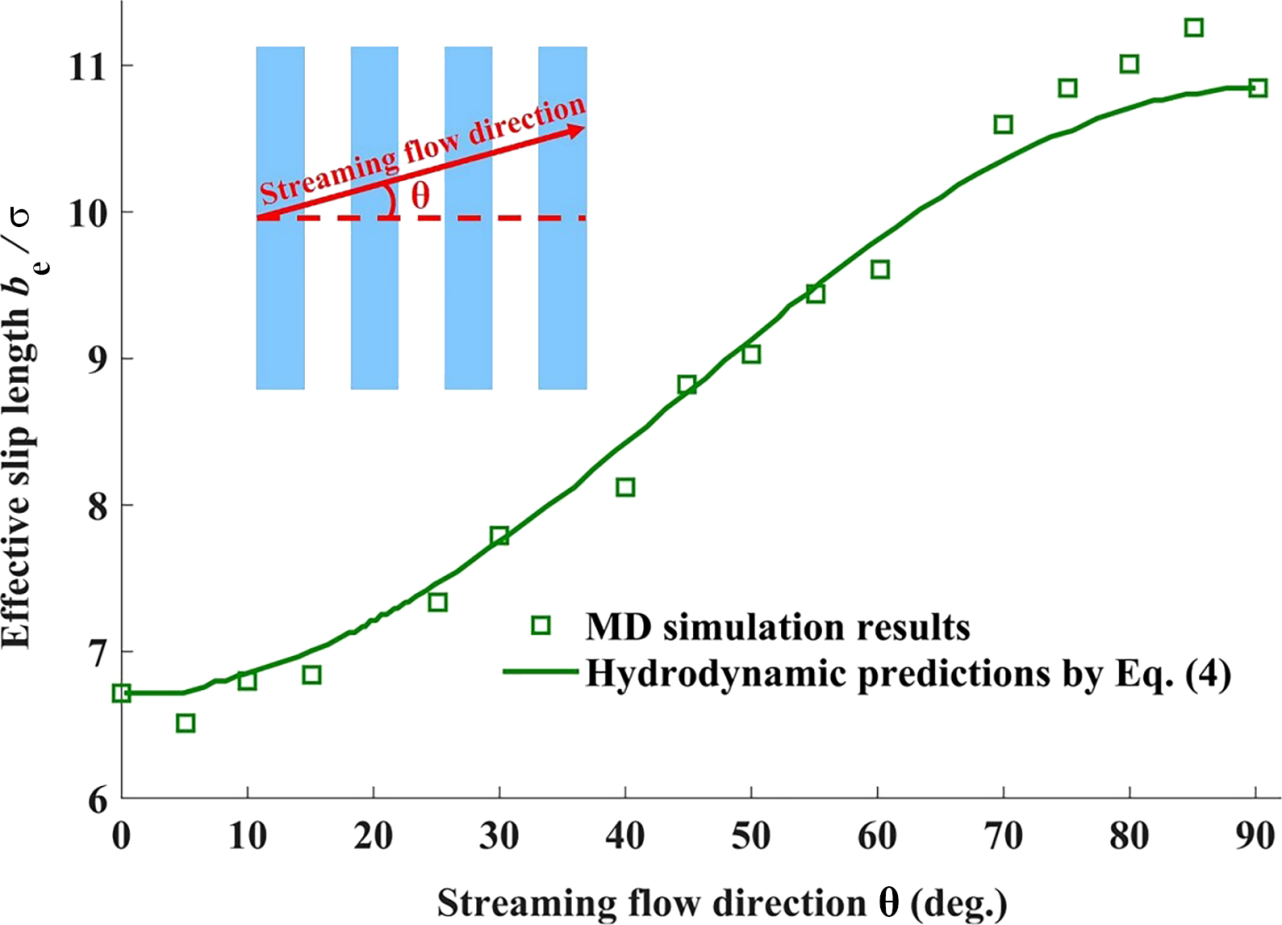
Variation of the effective slip length with streaming flow direction. The inset indicates the liquid flow direction relative to the stripe orientation. Figure 8 was redrawn from [111].

For periodic alternating stripes with different wettability, it was shown that the maximal and minimal effective slip length can be attained when the stripe orientation is parallel or perpendicular to the streaming flow direction, respectively. Especially, when the stripe widths for two-component textures are equal and smaller than the channel height, the variation of the effective slip length with the flow direction is given as follows [111]:


[4] $$b_{\text{e}}\left(\theta \right)=b_{1}\cos^{2}\theta +b_{2}\sin^{2}\theta ,$$

where θ indicates the streaming flow direction relative to the stripe orientation, *b*_1_ and *b*_2_ are the effective slip lengths when the liquid flows perpendicularly (θ = 0) and parallelly (θ = π/2) to the stripe orientation, respectively. As it can be seen from Figure 8, MD simulation results show good agreement with Equation 4 and the effective slip length monotonically increases with an increase in θ.

**Figure 9:**
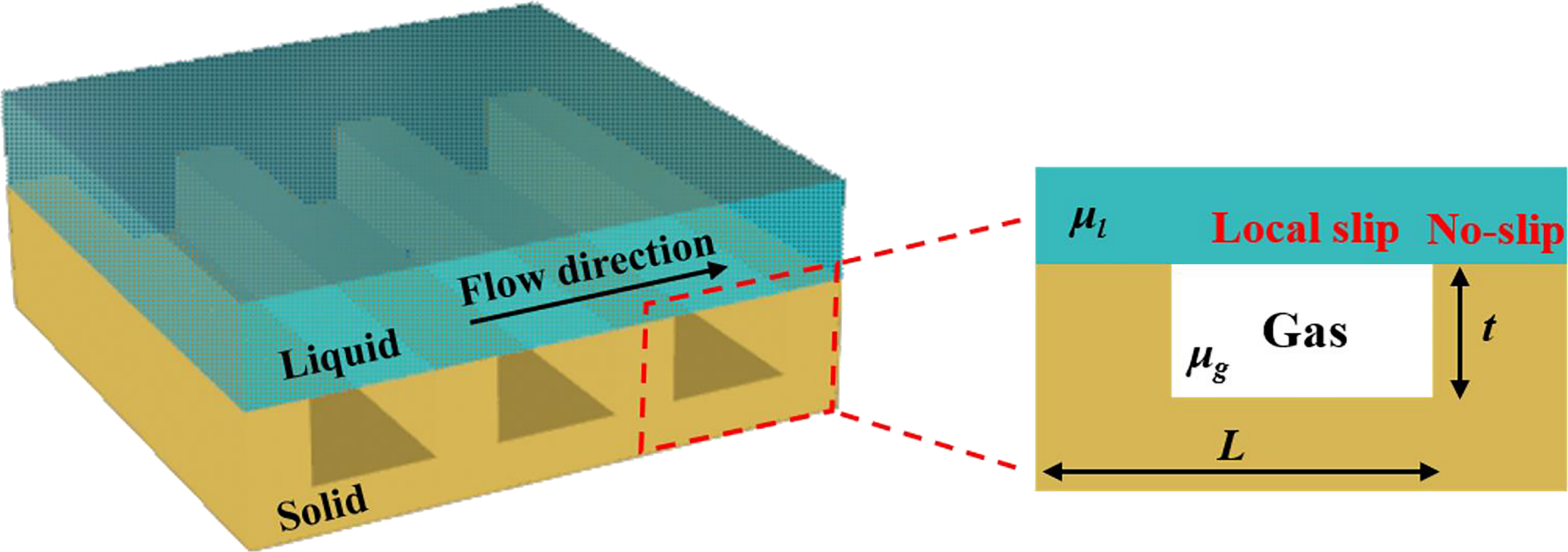
Sketch of the Cassie state of the liquid flow on a periodic structured surface.

**2.4.3 Gas bubbles.** Investigating the effect of gas bubbles on the effective slip length is also of great value especially in the design and optimization of superhydrophobic surfaces, which have lots of applications due to their superlubricating potential [112–114]. The liquid flow behavior on such surfaces can be described by the Cassie state, where the liquid only wets the top surfaces of the roughness with the gas pockets maintained in between the surface structures, as shown in Figure 9.

It is assumed that the no-slip boundary condition is still valid at the liquid–solid interface, while at the flat liquid–gas interface there is a finite local slip length (see Figure 9). The main difficulty to construct the effective slip boundary condition is that there is no general theory to describe the relationship between the local slip length at the liquid–gas interface and its parameters, such as the relief of the texture [115]. The classical “gas cushion” model gives an approximate value of the local slip length at the liquid–gas interface as follows [116]:


[5] $$b_{\text{g}}\approx \frac{\mu_{\text{l}}}{\mu_{\text{g}}}t,$$

where *b*_g_ is the local slip length at the liquid–gas interface, μ_l_ and µ_g_ are the viscosities of the liquid and gas, respectively, and *t* is the thickness of the gas layer. Typically, for the case of water flow over air at room temperature, we can approximately take the value of the local slip length at the liquid–gas interface as 50*t* [93]. It should be noted that the above equation is only valid for shallow grooves associated with the Cassie state for superhydrophobic surfaces. For periodic deep grooves on superhydrophobic Cassie textures or even for more complex 2D textures, the classical “gas cushion” model does not apply. Instead, a general gas cushion model proposed by Nizkaya et al. can better evaluate the local slip length at the liquid–gas interface [115].

### 3 Applications of nanofluidics with tunable slip length

#### 3.1 Drag reduction

Reducing drag is of great significance in many areas related to nanotechnology, such as nanotribology [117], nanomedicine [118], and electrokinetics [119] due to the low energy dissipation. For instance, it has been reported that the drag reduction might lead to the improvement of energy conversion efficiency from mechanical to electrical energy for the generation of streaming current induced by the pressure difference [119]. As stated in Section 1.1, the drag reduction is equivalent to the increase of slip length, which can be achieved by the modification of solid–liquid interfacial properties including surface wettability, surface roughness, and surface chemical composition. Therefore, the flow drag resistance can be reduced if the surfaces are appropriately engineered, or if the liquid properties are directly changed to induce a large slip length. Superhydrophobic surfaces are just such examples and liquids can experience extremely low drag resistance when flowing on those surfaces. Daniello et al. found that apart from the laminar flow regime, drag can still be reduced on superhydrophobic surfaces even in the turbulent flow regime, and at a given Reynolds number *Re*, the magnitude of drag reduction increases with the increase in feature size and spacing [120]. Furthermore, Srinivasan et al. reported that the dimensionless effective slip length is the key parameter that governs the drag reduction on superhydrophobic microstructures, and it is found to increase with the increase of the square root of the Reynolds number in the limit of high *Re* [121].

#### 3.2 Nanofiltration

**Figure 10:**
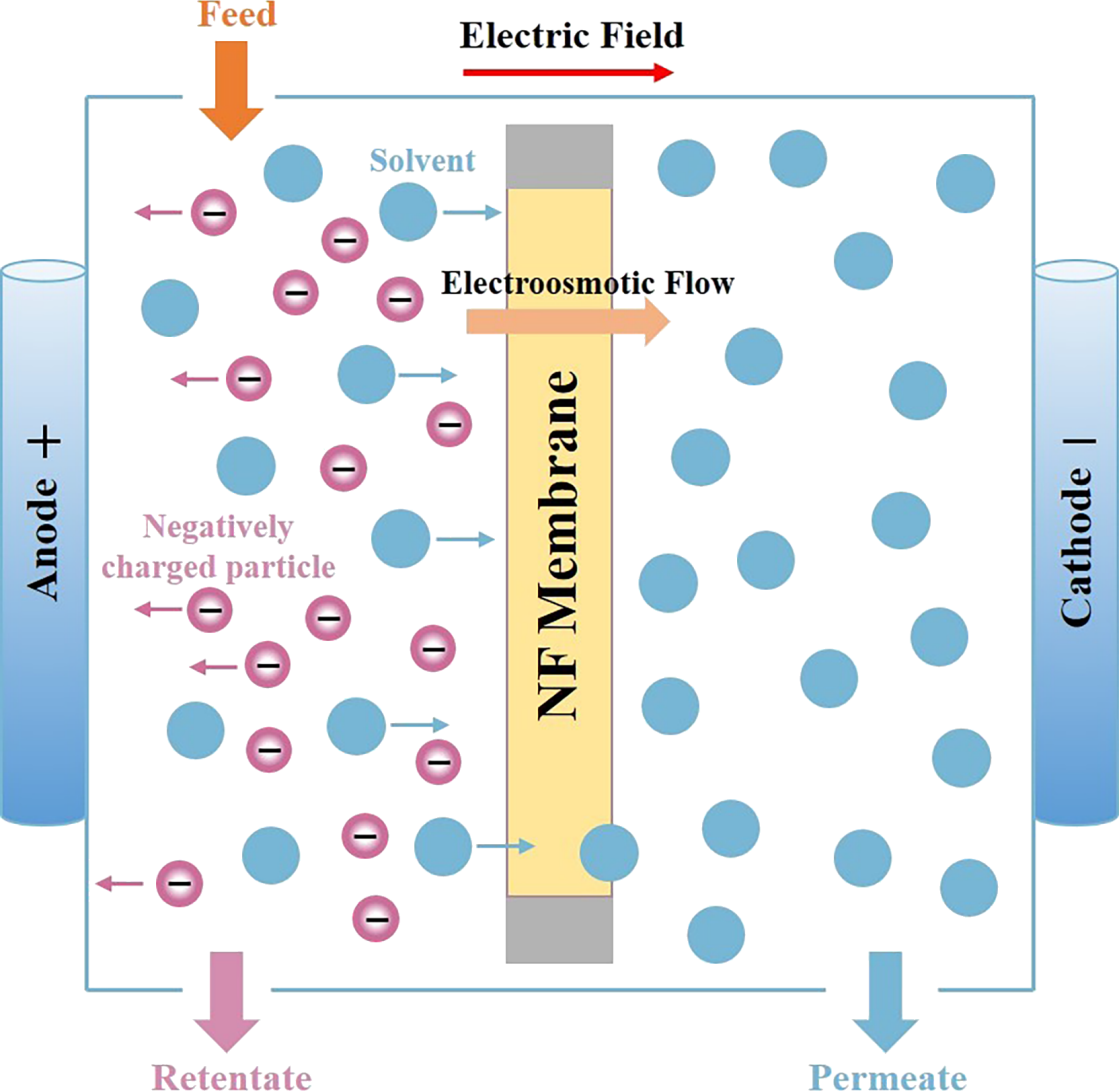
Schematic illustration of electro-osmosis process through a nanofiltration membrane.

As shown in Figure 10, electro-osmosis can play an important role in the area of nanofiltration, where membrane fouling is the main drawback that is frequently encountered in practical applications [122]. In addition, compared with hydrodynamic pumping, where the thickness of the EDL is much smaller than the channel geometrical length, the electro-osmotic pumping can induce large flow velocity, which can greatly enhance the permeate flux through the membrane [123]. Since improving the slip length can enhance the electro-osmotic velocity, the slip length should be taken into consideration when investigating the characteristics of electro-osmosis through membranes. Silkina et al. proposed a model which incorporates hydrodynamic slip and the mobility of surface charges to describe the motion of electro-osmosis. The approximate expressions for the electro-osmotic velocity can be derived even when the surface charge density is large [75]. When investigating the motion of an electrolyte solution driven by an external electric field, Celebi and Beskok compared a slip-modified theoretical model with the molecular dynamics simulation results. They found that the slip enhancement in the electro-osmotic flow is independent of the channel height, which can provide a theoretical basis for membrane fabrication of electrically assisted nanofiltration [80].

#### 3.3 Development of unconventional reservoirs

It is of great value to develop unconventional reservoir resources, including shale oil/gas and tight oil/gas due to the huge energy demand and large consumption of conventional energy. Different from conventional oil/gas reservoirs, the pore sizes in unconventional reservoirs range from micro- to nanometers [124]. The liquid flow in micro/nanopores can possess quite different characteristics from the macroscale liquid flow, and particularly the classical Darcy’s law may no longer be valid. It was demonstrated by many previous studies that in unconventional reservoirs with nanopores, the non-Darcy phenomena are ubiquitous for liquid flows [125–126]. Especially, there is a threshold pressure gradient (TPG) which should be overcome for the onset of the liquid flow. Nonetheless, the generation mechanism of TPG remains ambiguous and unresolved. One of the possible mechanisms is based on the existence of negative slip length and its variation. For example, combining with the negative slip length, Song et al. proposed a single-phase nonlinear seepage model and successfully explained the nonlinear flow characteristics of low permeability reservoirs [127]. Therefore, the negative slip length and its variation could significantly affect the transport behavior of nanoconfined liquid, and hence should be taken into account when evaluating the production of unconventional oil/gas reservoirs.

## Conclusion

In this review, some concepts including the definition and categories of slip length have been presented. Then, the effects of interfacial properties on slip length as well as the potential applications of tunable slip lengths for nanoscale slip boundary conditions have been analyzed and discussed.

To sum up, the solid–liquid interfacial properties can dramatically affect the slip boundary conditions for a nanoconfined liquid flow. Depending on the surface wettability, the slip length can be positive or negative. A negative slip length could exist when a liquid flows on a strong hydrophilic surface, and its magnitude can vary with the external driving force. Furthermore, for an electro-osmotic flow, surface charge can also influence the slip length, and with the increase in surface charge, the slip length decreases. In addition, the surface roughness has also a direct impact on the effective slip length, and the variations of the effective slip length with the amplitude of surface roughness are different depending on whether there is true slip length. Moreover, the effective slip length can also be affected by the composition of surface textures, including isotropic textures, anisotropic textures, and gas bubbles.

Finally, the tunable slip length can be of great value in many areas, including drag reduction, nanofiltration, and in the development of unconventional reservoirs. For future works, the mechanism of negative slip length variation should deserve more attention. In addition, more applications of nanofluidic systems with tunable slip length in areas related to nanotechnology, such as ion separation and drug discovery, should be investigated and developed.
